# Enhancement of *in vitro *interleukin-2 production in normal subjects following a single spinal manipulative treatment

**DOI:** 10.1186/1746-1340-16-5

**Published:** 2008-05-28

**Authors:** Julita A Teodorczyk-Injeyan, H Stephen Injeyan, Marion McGregor, Glen M Harris, Richard Ruegg

**Affiliations:** 1 Division of Graduate Education and Research, Canadian Memorial Chiropractic College, Canada; 2 Division of Foundational and Professional Education, Canadian Memorial Chiropractic College, Canada; 3 Division of Clinical Education, Canadian Memorial Chiropractic College, Canada

## Abstract

**Background:**

Increasing evidence supports somato-visceral effects of manual therapies. We have previously demonstrated that a single spinal manipulative treatment (SMT) accompanied by audible release has an inhibitory effect on the production of proinflammatory cytokines in asymptomatic subjects. The purpose of this study is to report on SMT-related changes in the production of the immunoregulatory cytokine interleukin 2 (IL-2) and to investigate whether such changes might differ with respect to the treatment approach related to the presence or absence of an audible release (joint cavitation).

**Methods:**

Of 76 asymptomatic subjects, 29 received SMT with cavitation (SMT-C), 23 were treated with SMT without cavitation (SMT-NC) and 24 comprised the venipuncture control (VC) group. The SMT-C and SMT-NC subjects received a single, similar force high velocity low amplitude manipulation, in the upper thoracic spine. However, in SMT-NC subjects, positioning and line of drive were not conducive to cavitation. Blood and serum samples were obtained before and then at 20 and 120 min post-intervention. The production of IL-2 in peripheral blood mononuclear cell cultures was induced by activation for 48 hr with Staphylococcal protein A (SPA) and, in parallel preparations, with the combination of phorbol ester (TPA) and calcium ionophore. The levels of IL-2 in culture supernatants and serum were assessed by specific immunoassays.

**Results:**

Compared with VC and their respective baselines, SPA-induced secretion of IL-2 increased significantly in cultures established from both SMT-C and SMT-NC subjects at 20 min post-intervention. At 2 hr post-treatment, significant elevation of IL-2 synthesis was still apparent in preparations from SMT-treated groups though it became somewhat attenuated in SMT-NC subjects. Conversely, IL-2 synthesis induced by TPA and calcium ionophore was unaltered by either type of SMT and was comparable to that in VC group at all time points. No significant alterations in serum-associated IL-2 levels were observed in any of the study groups.

**Conclusion:**

The present study demonstrates that, the *in vitro *T lymphocyte response to a conventional mitogen (SPA), as measured by IL-2 synthesis, can become enhanced following SMT. Furthermore, within a period of time following the manipulative intervention, this effect may be independent of joint cavitation. Thus the results of this study suggest that, under certain physiological conditions, SMT might influence IL-2-regulated biological responses.

## Background

Two of the most intriguing controversies surrounding the chiropractic profession are: 1) the biological mechanisms associated with spinal manipulation [1,2], and 2) the clinical impact of the audible release that comes from a high velocity, low amplitude force directed to a spinal joint [3]. The research reported here adds to the body of knowledge in each of these important areas: samples of cell culture supernatants obtained in the course of an earlier study examining the effect of spinal manipulation on selected parameters of the immune response [4] were evaluated for changes in the capacity for production of the immunoregulatory cytokine, interleukin 2 (IL-2), pre- and post-intervention, as well as, between manipulation characterized by an audible release (cavitation) and manipulation without audible release.

The current model of spinovisceral (somatoautonomic) reflex provides a link between spinal manipulation, the autonomic nervous system (ANS) and various visceral functions [5]. Although experimental studies have demonstrated that somatoautonomic reflex effects are evident in various non-musculoskeletal systemic reactions [6] studies in humans are still limited, particularly with respect to the effect of spinal manipulative therapy (SMT) on the integrated responses of the nervous and immune systems. Clarification of such a relationship can have clinical implications in the area of manual interventions in pursuit of somato-visceral outcomes. Our recent studies on asymptomatic subjects receiving a single high velocity low amplitude spinal manipulation (HVLA), characterized by cavitation (audible release), in the thoracic spine, demonstrated suppression of the *in vitro *production of two proinflammatory cytokines, tumor necrosis factor α (TNFα) and interleukin 1β (IL-1β) [4] relative to subjects receiving a high velocity, low amplitude force applied to the thoracic spine, without cavitation. Earlier studies by Brennan et al. [7] suggested that a series of high force, HVLA manipulations of the lumbosacral spine of low back patients, as well as asymptomatic controls, did not result in quantitative changes of lymphocyte subpopulations in peripheral blood samples collected from either group. To our knowledge, however, the effect of SMT (of any type) on the functional activity of these cells, including their capacity for cytokine production, has not been studied.

IL-2, originally identified as a T-cell growth factor, is now recognized as a pivotal cytokine in T-cell dependent immune responses [8]. An increasing body of evidence indicates also that IL-2 plays a major role in the development, maintenance and survival of regulatory T cells warranting its critical importance in the induction and sustenance of immune tolerance [9]. Determinations of *in vivo *(serum) IL-2 levels have been applied in clinical studies in which systemic activation of T-cells is suspected [10]. On the other hand, assessment of the *in vitro *production of this cytokine by mitogen-stimulated human cell cultures has been long used as a standard procedure for scrutinizing the capacity of T lymphocytes to become activated [11]. Thus, both *in vivo *and *in vitro *determinations of IL-2 have been utilized in this investigation. Furthermore, the *in vitro *induction of IL-2 synthesis may be achieved either via mitogen-related transmembrane signaling or by a chemical stimulation which bypasses such requirements. Both methods have been utilized in the present study.

It has been suggested that cavitation (the audible release) associated with HVLA is what "truly" distinguishes SMT from other manipulation-type modalities [12]. The intervention methods used in this investigation are consistent with this notion. That is, HVLA forces were applied to some subjects with the intent of producing an audible release, and in other subjects with the intent of not producing a release. Controversy remains as to whether cavitation results in any clinically relevant differences [13,14]. Studies have not yet been conducted to determine if differences due to cavitation exist at a cellular/molecular or immunological level. This investigation begins such an exploration.

## Methods

### Subjects

All subject-handling procedures were approved by the Canadian Memorial Chiropractic College (CMCC) Research Ethics Board. The present study was part of a larger investigation in which blood samples were obtained from 82 age- and sex-matched participants to test for changes in different parameters of the immune response following a spinal manipulative intervention. Subjects naive to spinal manipulation were recruited through announcements and advertisement from CMCC students within the first two weeks of entering their first year of study, first year naturopathic students, or the community at large. In the present study, for determination of IL-2 levels, samples were available from 76 of the subjects (Table 1).

**Table 1:** Demographic data of subjects

**Group**	**Age (years)**	**Sex (female/male)**
VC, n = 24	24.1 ± 1.5	15/9
SMT-NC n = 23	25.3 ± 1.21	13/10
SMT-C n = 29	24.8 ± 1.75	15/14

VC, venipuncture control; SMT-NC, spinal manipulative therapy-no cavitation;

SMT-C, spinal manipulative therapy-cavitation.

Details of the experimental design and protocol have been described previously in the context of investigating changes in proinflammatory cytokine production in the same subjects [4]. Briefly, subjects were accepted into the study if they had not received previous chiropractic treatments regularly and had not received any manipulative treatments for a minimum of the previous 6 months; and in whom the study clinician was able to identify a restricted motion segment in the upper thoracic spine. Patients were excluded if they had current aches or pains due to any complaints, recent or current strain/sprain type injuries, infections or fracture, a history of neoplasm or other diseases involving the immune system, or history of recent emotional or physical trauma. They were screened by a single clinician, using motion and static palpation, for notional restrictions in segmental motion in the upper thoracic spine (T1–T6). Subjects in whom no restrictions could be identified were dismissed from the study.

Those accepted into the study were randomly assigned to one of 3 groups: spinal manipulation with cavitation (SMT-C), spinal manipulation without cavitation (SMT-NC) or venipuncture control (VC). SMT consisted of a single bilateral hypothenar push (Carver Bridge) adjustment [15] applied to the involved vertebral segment and with sufficient force so as to produce joint cavitation as judged by the treating clinician. For SMT-C force was applied to the restricted segment in a direction normal to the surface of that region of the thoracic spine, and the thrust was delivered downwards albeit with a cephalad component inherent to the Carver Bridge procedure. The procedure for SMT-NC consisted of an identical set-up using similar force but with positioning and line of drive essentially in an extreme cephalad direction with the intention of avoiding joint cavitation. In two instances, where cavitation did occur, subjects were designated to the SMT-C group. In an earlier study using the same subjects, we had referred to this latter group as having received a sham manipulation [4]. Subjects in the VC group received only the set up "joint loading" without the application of thrust.

Both heparinized and non-heparinized blood samples were obtained from all subjects prior to intervention and then at 20 min and 2 hr post-intervention. The former were used for studies of the *in vitro *production of IL-2, while systemic (in *vivo*) levels of IL-2 production were determined in sera derived from non-heparinized blood samples. A coding system was used that allowed blinding of the venipuncturist and laboratory investigators to the subjects in all three groups. All samples to be examined were thawed immediately before testing.

### In vitro IL-2 levels

The in *vitro *studies were carried out to investigate if the capacity for an inducer-related IL-2 production by peripheral blood mononuclear cells (PBMCs) isolated from heparinized blood samples was altered as a result of SMT. In addition, because, PBMCs can synthesize low levels of IL-2 spontaneously (that is without stimulation) the amounts of IL-2 synthesized in cultures cultivated in the absence of inducer were also determined.

### Peripheral blood mononuclear cell (PBMC) culture system

PBMCs were isolated by density gradient centrifugation on Ficoll-Hypaque according to the protocol recommended by the producer (Pharmacia Biotech, Uppsala, Sweden). Cells were then prepared for *in vitro *studies essentially as described previously [10]. Tissue culture medium (TCM) used throughout the study consisted of RPMI 1640 supplemented with a pretested batch of fetal calf serum (10%v/v), 2 mM L-glutamine, 5 × 10^-5 ^M 2-mercaptoethanol (Sigma, ST. Louis, MO) and antibiotics. All solutions were freshly prepared from stocks by diluting with TCM to desired concentrations. PBMCs were suspended in TCM at a concentration of 1 × 10^6^/ml and aliquoted, in duplicate, into tissue culture tubes for cultivation.

### Induction of IL-2 production

IL-2 synthesis in PBMC cultures was induced by two different methods. The conventional mitogen, Staphylococcal protein A (SPA) [16], (Pharmacia Chemicals, Uppsala, Sweden) was used at a final concentration of 10 μg/ml. In parallel cultures, IL-2 synthesis was induced by the application of the combination of phorbol ester 12-o-tetradecanoyl-phorbol-13-acetate (TPA) and calcium ionophore A23187 [17]. A23187 (Sigma) was dissolved in ethanol and used at a concentration of 200 ng/ml and TPA (Sigma) was prepared in DMSO, and used at the final concentration of 3 × 10^-8 ^M. Cultures were maintained for 48 hr at 37°C in a humidified 5% CO_2 _incubator. At the conclusion of the incubation period, culture supernatants from each subject were pooled, aliquoted and frozen at -78°C until further analysis.

### The assay procedure

The levels of IL-2 in supernatants from PBMC cultures were determined by specific enzyme-linked immunosorbant assays (ELISA) using DuoSet ELISA development system for natural and recombinant human cytokines (R&D Systems, Minneapolis, MN). Briefly, Immulon 4 HBX, flat bottom microtiter plates (Thermo Labsystems, Franklin, MA) were coated with a predetermined concentration of mouse anti-human IL-2 antibody. After washing and blocking the wells with bovine serum albumin, duplicate dilutions of standards (defined amounts of a human recombinant IL-2) or of the test supernatants were added to the wells and incubated for 2 hours. Plates were then washed and incubated with a specific detection antibody (biotinylated goat anti-human anti-IL-2 antibody). After 20 min incubation and multiple washing, the plates were incubated for 20 min with streptavidin-HPR solution and then with substrate solution (mixture of H_2_O_2 _and tetramethylbenzidine). Following the development of colour, the absorbance was measured at 450 nm using an automated microplate reader (ELx800, Bio-Tech Instruments, Inc. Winooski, Vermont, USA). Concentrations of IL-2 were calculated from the linearized (best fit) standard curve determined by regression analysis. Each culture supernatant was tested at least twice at 2–4 different dilutions in order to ascertain if determinations of IL-2 were within the parameters of the standard curve.

### In vivo (serum) IL-2 levels

The *in vivo *(systemic) production of IL-2 was examined by determination of the serum content of IL-2. Non-heparinized blood (5 ml) was drawn into a separation tube and placed in ice. After 1 hour, these specimens were centrifuged at 4°C and sera were aliquoted to be frozen at -78°C until use. Just prior to assays being done, samples were thawed, diluted with phosphate buffered saline (PBS) supplemented with 10% FCS, and processed by ELISA as described above.

### Enumeration of cells

A quantitative analysis of peripheral mononuclear cells was performed as described previously [18].

### Statistics

Significant inter-individual variability in the capacity of human cytokine production is typical even though within individual production is relatively constant [19,20]. Normality tests of sample data indicated that the distribution of baseline and both early (20 minute) and late (2 hour) post-intervention measures were within tolerable limits [21]. Analysis of covariance (ANCOVA), was therefore used to assess for differences between intervention conditions (venipuncture control, HVLA force with audible release and without) at each outcome time, while considering baseline measurement as the covariate to account for any initial between-group differences. Stata SE8 was used to perform the ANCOVAs.

## Results

### IL-2 synthesis in vitro

Constitutive (spontaneous) and inducer-related synthesis of IL-2 were both examined in PBMC cultures. Constitutive synthesis of IL-2 was determined in supernatants from unstimulated (inducer-free) cultures cultivated in parallel to inducer-activated preparations. Only cultures for which a complete set of data was available were included in the final analysis. Routine flow cytometry (FACS) examination of cell surface markers ascertained that the numbers of IL-2 producing lymphocytes were comparable in all PBMC preparations.

### Constitutive (spontaneous) production of IL-2

Seven (2 each in VC and SMT-NC and 3 in SMT-C group) of 76 subjects investigated in this study demonstrated no detectable IL-2 synthesis in unstimulated PBMC cultures both prior to and post-intervention. The levels of IL-2 released spontaneously in cultures from the remaining subjects showed no significant alterations over the study period. The mean levels of synthesis of IL-2 in these preparations ranged from 29–41 pg/ml and were comparable in all study groups before (baseline) and at 20 min and 2 hr post-intervention. This suggested that neither the venipuncture procedure alone nor manipulative intervention (SMT-NC or SMT-C) altered the state of activation of circulating IL-2-producing lymphocytes.

### IL-2 production induced by SPA

#### Twenty minutes post-intervention

**Figure 1:**
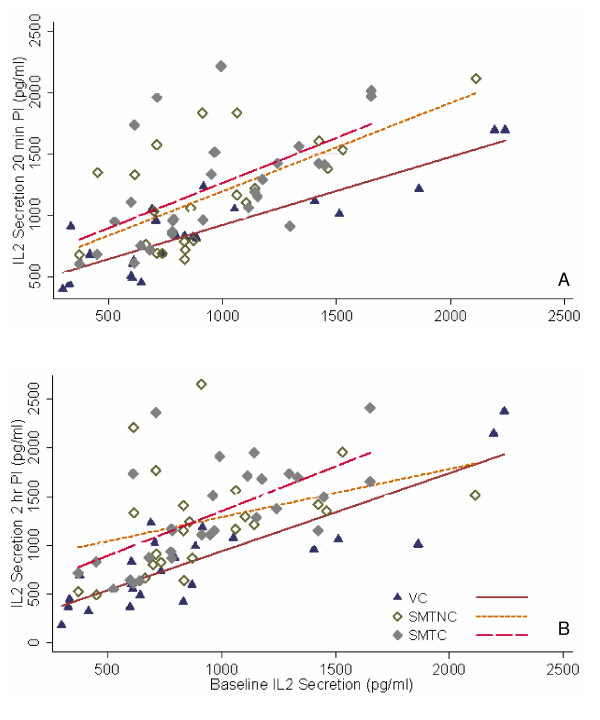
**SPA-induced IL-2 secretion in cultures established 20 minutes (A) and 2 hours (B) post-intervention (PI)**. PBMC cultures were activated with SPA (10 μg/ml) at initiation and incubated at 37°C for 48 hr. The concentration of IL-2 (pg/ml) produced *in vitro *by cells from a subject at 20 min (A) and 2 hr (B) post intervention is plotted against the concentration of IL-2 produced at baseline by the same subject. Each point represents a subject for whom a complete set of data was available. Comparison of best fit lines revealed statistically significant differences between SMT-C (spinal manipulative therapy with cavitation, n = 26) and VC (venipuncture, n = 21) as well as SMT-NC (spinal manipulative therapy without cavitation, n = 23) and VC both for 20 min and 2 hr post intervention (see text for details).

The production of IL-2 increased in cultures from SMT-C as well as SMT-NC subjects relative to baseline and venipuncture controls 20 minutes following the manipulative interventions. Analysis of covariance results revealed statistically significant differences between the groups at this time point (F = 7.67, p = 0.001). In particular, post-hoc analysis determined a statistically significant difference between the venipuncture control (VC) and the group that received the manipulation with an audible release (cavitation – SMT-C) (F = 14.30, p = 0.000). A statistically significant difference was also found between the VC and the group that received a manipulation without an audible release (no cavitation – SMT-NC) (F = 8.01, p = 0.006). No difference was found in post-hoc comparison between the SMT-C and SMT-NC (F = 0.61, p = 0.436). These results are represented graphically in Figure 1A.

#### Two hours post-intervention

An increase in IL-2 production was also apparent in cultures from both SMT-C and SMT-NC subjects relative to baseline and venipuncture controls. Analysis of covariance revealed a statistically significant difference between groups at two hours after the intervention (F = 7.51, p = 0.001). Once again, post-hoc analysis showed this difference to be between the VC and SMT-C (F = 12.99, p = 0.001) as well as between the VC and SMT-NC (F = 9.54, p = 0.003). Again, no difference was found between the SMT-NC and SMT-C (F = 0.11, p = 0.737) (Fig 1B). Interestingly, the graphical representations in Figure 1 show that while statistically, the results from two hours are almost identical to those at 20 minutes, some degradation of the line associated with SMT-NC already appears to have occurred.

### IL-2 production induced by TPA

#### Twenty minutes post-intervention

**Figure 2:**
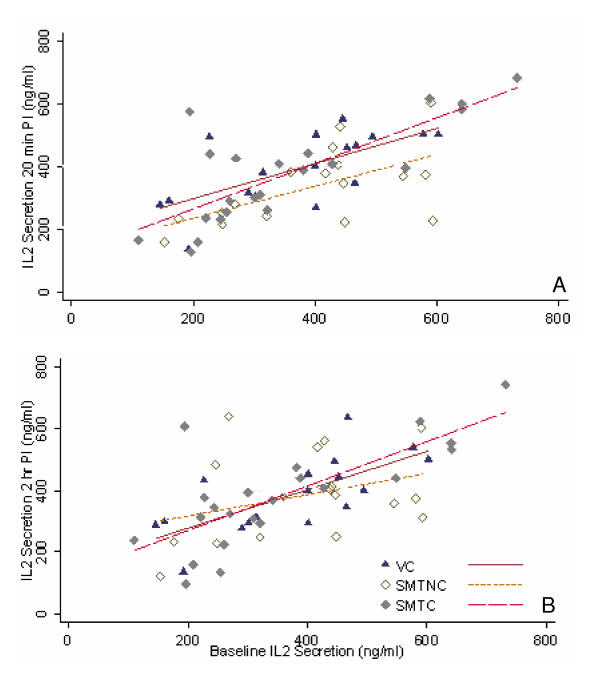
**Phorbol ester (TPA)-induced IL-2 secretion in PBMC cultures established 20 minutes (A) and 2 hours (B) post-intervention (PI)**. PBMC cultures were activated with the combination of TPA (3 × 10^-8 ^M) and calcium ionophore A23187 (200 ng/ml) at initiation and incubated at 37°C for 48 hr. IL-2 levels were determined in culture supernatants by specific ELISA. Each point represents the concentration of IL-2 (ng/ml) produced *in vitro *by cells from a subject at 20 min (A) and 2 hr (B) post intervention plotted against the concentration of IL-2 produced at baseline by the same subject. Each point represents a subject for whom a complete set of data was available. Comparison of best fit lines revealed no statistically significant differences between SMT-C (spinal manipulative therapy with cavitation, n = 22) and VC (venipuncture, n = 16) as well as SMT-NC (spinal manipulative therapy without cavitation, n = 17) and VC both for 20 min and 2 hr post intervention (see text for details).

Analysis of covariance of IL-2 secretion induced by the combination of TPA and ionophore revealed that at 20 minutes post-intervention there was no significant difference between the three groups (F = 2.59, p = 0.0846). The data are represented graphically in Figure 2A.

#### Two hours post-intervention

Analysis of covariance under this set of experimental conditions (two hours after intervention) was consistent with the results at 20 minutes. No statistically significant difference in IL-2 secretion was found between the three groups (F = 0.22, p = 0.8023), (Fig 2B).

### IL-2 synthesis in vivo

Determinations of systemic (*in vivo*) release of IL-2 were carried out using serum samples collected at the same time as PBMCs used for the *in vitro *studies described above. As resting lymphocytes do not produce IL-2, sera from normal individuals contain usually very little or no detectable IL-2. At baseline, IL-2 was detected in serum from only 5/24 subjects in VC, 6/23 in SMT-NC and 9/29 in the SMT-C treated subjects. The mean values of detectable serum IL-2 levels were comparable in all study groups (15–21 pg/ml), and remained essentially unaltered during the post-intervention period.

## Discussion

The biological mechanisms associated with spinal manipulation are poorly understood [1]. However, evidence has been accumulating that somatovisceral effects may result from a spinal manipulative therapy [6]. We have previously demonstrated that a single spinal HVLA manipulation, characterized by cavitation and intended to mobilize a small joint "fixation" in the upper thoracic spine in asymptomatic subjects, has a suppressing effect on proinflammatory cytokine synthesis by PBMCs *in vitro *[4]. Earlier studies have demonstrated increased activity of the innate immune response components following a single SMT in the upper T-spine of asymptomatic subjects [22,23]. However, parameters of specific immunity have not been investigated with a view of determining possible alterations in lymphocyte functions. In the present study we have demonstrated that following a spinal manipulative intervention the *in vitro *synthesis of IL-2 increases significantly, both with and without cavitation, for at least the short term (20 min post-SMT). These results are important from two different perspectives: a) the effect (increase) may be independent of joint cavitation and b) the response is evident in mitogen (SPA)-activated cultures (Figs 1A, 1B) but not those stimulated by the combination of TPA and calcium ionophore (Figs 2A, 2B)

The design of our study does not allow for speculation as to the mechanism(s) involved in the transduction of a putative effect of the manipulative thrust to the cellular/molecular level *in vivo*. However, the *in vitro *application of two inducers dissimilar with respect to the route by which the activating signal was provided allows for an insight into the mechanism of SMT action in our experimental model system. SPA is suggested to induce preferential activation of CD4+ (Th1) cells through its ability for cross-linking with the MHC class II molecules on the surface of antigen presenting cells APC [24]. The application of phorbol ester and calcium ionophore by-passes this requirement and mobilizes molecular mechanisms of IL-2 synthesis directly [25,26]. The results of the present study demonstrate clearly that manipulative intervention provided by spinal manipulation of the thoracic spine, results in a significant augmentation of IL-2 secretion *in vitro *induced by SPA.

The increased IL-2 synthesis by PBMCs was not accompanied by a systemic (*in vivo*) release of IL-2 in response to any of the interventions. Thus, at baseline, serum IL-2 was detected in a small number of subjects and did not increase post-manipulation. Similarly, constitutive (spontaneous) *in vitro *secretion of IL-2 remained consistently comparable in all study groups. It is therefore unlikely that the observed increments in SPA-induced production of IL-2 ensued from *in vivo *(systemic) pre-activation of circulating peripheral blood T lymphocytes. Rather, it is possible that the manipulative interventions had a priming effect on cells/factors *in vivo *with a subsequent enhancing effect on the interaction between antigen presenting cells, principally dendritic cells (DCs), and T lymphocytes. Such improved interaction has been shown to be mediated by another cytokine, interleukin 15 (IL-15) resulting in increased IL-2 production by T lymphocytes, such as observed in our study. Interestingly, IL-15 which is highly expressed in neural tissues and skeletal muscles [27] is released in large quantities following physical stress [28,29].

In the present study, it is not known if any of the procedures (including the "laying on of the hands", the manipulative thrust) to which participants were subjected to, were sufficiently stressful to cause an increase in the systemic output of IL-15. Venipuncture controls were examined and touched by the clinician in a manner comparable to the SMT groups, making it unlikely that the observed effect on IL-2 synthesis is due to "laying on of the hands" or anticipation-related stress. What remains common to both SMT-C and SMT-NC is the manipulative force. It has been reported that a threshold force may be required in order to attain SMT-related physiological changes [23]. Manipulative forces were not measured in this study and we cannot stipulate as to the magnitude of force to which participants in the SMT-NC or SMT-C groups were subjected. However, it is feasible that manipulative forces and intervention-related stress increased the systemic levels of IL-15 in both SMT-C and SMT-NC groups. This could improve DC interaction with T lymphocytes and increase IL-2 production. Finally, IL-15-activated DCs themselves produce IL-2 in considerable quantities [30]. The process of DC stimulation resulting in IL-2 synthesis requires the presence of both T lymphocytes and microbial product. [31]. In the present study, these conditions were met in SPA- but not TPA-stimulated cultures of PBMCs, which overall, showed no alterations in IL-2 production (Figs 2A, 2B).

The observed IL-2 increase might also be due to an increase in the number of DCs. Earlier studies have revealed no quantitative changes in T cells; however, enumeration of PBMCs expressing the DC phenotype was not done. It has been demonstrated that physical stress increases the number of DC cells in the circulation [32]. Thus, an increase in the number of IL-2 secreting DCs in the PBMC preparations examined after the intervention, might explain the observed increase in IL-2 production in cultures derived from SMT-C and SMT-NC – treated subjects. Future studies of possible alterations in DC numbers and biological responses following manipulative interventions should yield informative results in this regard.

The results of this study indicate that the increase in IL-2 synthesis by PBMCs due to a manipulative intervention was independent of joint cavitation when considered in the 20 minute time frame following SMT. Statistical analysis of the data for 2 hours post intervention revealed similar results. However, in the absence of cavitation there was an attenuation of the effect (Fig 1B) suggesting that joint cavitation may have a longer lasting effect. It may be argued that increasing the number of participants in the study groups might have delineated differences between the SMT-C and SMT-NC groups more clearly. However, the p values (0.006 or less) revealed by the statistical analysis indicate that the study had adequate power. Nevertheless, future studies will benefit from reproducing these results with similar or better statistical power, and more importantly, exploring the time course of IL-2 synthesis, by making determinations over a longer period (e.g. 1, 2, 3, and 24 hours) following SMT.

While there is controversy around the topic of the clinical significance of cavitation [13,14,33] the results reported here, as well as earlier findings of cavitation-dependent-suppression of proinflammatory cytokines in response to SMT [4] suggest, that depending on the time-frame of determinations and/or type of outcomes investigated, cavitation may be an important qualifier of a successful HVLA thrust. A clear association between manipulation of the lumbar spine and zygapophyseal joint gapping has been demonstrated by use of magnetic resonance imaging in normal subjects [34]. However, a relationship between joint gapping and cavitation remains to be established. It has been suggested that while cavitation may be a reliable indicator of joint gapping, the mechanical effects of spinal manipulation may not be directly related to cavitation [2,12,33]. Flynn et al. [14] found that an "audible pop" did not change the outcome for sacroiliac region manipulation in patients with non-radicular low back pain. In their study all participants were manipulated similarly and were grouped into "audible pop" and "no audible pop" post manipulation. However, in this type of design it is possible that the perceived absence of an "audible pop" is due the fact that articular release may occur without being audible [35]. In the present study the SMT-NC group received a manipulative thrust designed not to produce cavitation. Nevertheless, we realize that in order to make an assertion with respect to the occurrence or absence of cavitation more rigorous means need to be employed. To this end the use of accelometry [36] in future studies will be useful. In addition, quantification of manipulative forces will shed further light on the relationship between IL-2 synthesis and HVLA with or without cavitation.

## Conclusion

A single HVLA manipulation to the thoracic spine of asymptomatic subjects causes a significant enhancement in IL-2 secretion by PBMCs *in vitro*. This increase is independent of joint cavitation at least for the short term. However, the duration of the effect appears to be time-restricted as evidenced by the decline of IL-2 levels 2 hours post-intervention in the non-cavitating group. These observations suggest that under certain physiological conditions, SMT could influence, directly or indirectly, IL-2-regulated biological responses. The results provide impetus for further investigations in order to clarify the relationship between HVLA manipulation with or without cavitation and the synthesis of IL-2 and other immunoregulatory mediators.

## Competing interests

The authors declare that they have no competing interests.

## Authors' contributions

JTA contributed to the design of the study, was responsible for all laboratory procedures, analysis of data, and contributed to the writing of the manuscript.

HSI contributed to the design of the study, was responsible for subject recruitment and coordination of the study, analysis of data, and contributed to the writing of the manuscript, MM performed the statistical analysis of the data and contributed to the writing of the manuscript, GMH contributed to data analysis and writing of the manuscript, RR contributed to the design of the study and was the study clinician. All authors have read and approved the final version of the manuscript.
